# Determinants of Change in Fertility among Women in Rural Areas of Uganda

**DOI:** 10.1155/2019/6429171

**Published:** 2019-12-19

**Authors:** Paulino Ariho, Abel Nzabona

**Affiliations:** ^1^Department of Sociology and Social Administration, Kyambogo University, Uganda; ^2^Department of Population Studies, School of Statistics and Planning, Makerere University, Uganda

## Abstract

Fertility among rural women in Uganda continues to decline. Studies on fertility in Uganda have focused on the overall fertility in the country. In this study, we focus on determinants of change in fertility among rural women in Uganda using a multivariate Poisson decomposition technique to quantify the contribution of changes in the socioeconomic and demographic composition of women which we also refer to as the characteristic effects and changes in their fertility behavior (the coefficients' effects or risk of childbearing) to the overall reduction in fertility among women in rural areas during the 2006–2016 period. The “characteristics effects” are used to mean the effect of changing composition of women by the socioeconomic and demographic characteristics between 2006 and 2016. On the other hand, fertility behavior also presented as coefficients' effects mean changes in the risk or likelihood of giving birth to children by the rural women between the two survey years. Our findings indicate that the mean number of children ever born (MCEB) reduced from 4.5 to 3.9 in 2006 and this reduction was associated with both the changes in composition of women and fertility behavior. The composition of women contributed to 42% while the fertility behavior contributed to 58% of the observed reduction. The education level attained and the age at first sex showed significant contributions on both components of the decomposition. The observed decline in fertility is largely associated with the variation in the risk of childbearing among the rural women. The variation in the risk of childbearing by education and age at first sex of the rural women showed to be the biggest contribution to the observed change in fertility. Continued improvements in access, attendance, and completion of secondary schools by women in rural areas will be the key drivers to Uganda's overall transition to low fertility. Furthermore, with improved access to mass media in the rural areas, there can be changes in attitudes and large family size preferences which can create a conducive environment for the utilization of family planning services in the rural communities. Efforts should therefore focus on applying appropriate methods to deliver packaged family planning messages to these communities.

## 1. Introduction

African fertility has been higher than in other developing countries in the past several decades and this persistent high fertility has been linked to the low level of socioeconomic development relative to other developing regions [1]. Although the African fertility has been exceptionally different from that of other regions, Africa has remarkable fertility diversity which is in fact increasing with the fertility situation ranging from pretransitional to replacement fertility[2].

It is generally agreed that fertility rates have declined globally but debate on why fertility rates have declined remains significant [3]. Many theories and frameworks explaining fertility change have been propounded. The major explanation of fertility change and dynamics has its origins in demographic transition theory (DTT) first developed by Thompson in 1929 and Notestein in 1945 [4]. This theory attributes fertility decline to changes linked to industrialization and urbanization that initially produce a decline in mortality and later fertility decline. This theory has however been found to be weak in explaining fertility transition in less urbanized and industrialized countries. The wealth flows theory propounded by Caldwell in a 1976 essay attributes fertility decline to the nucleation of the family which may be triggered by either economic or cultural forces [5]. This theory was based on studies conducted in West Africa (Ghana and Nigeria) where extended families were strong and lineage elders were likely to benefit from high fertility [5]. The ideational theory of fertility decline attributes the timing of fertility transition to the diffusion of information and new social norms about birth control [6]. Although this theory adds an important element to earlier theories, Cleland and Wilson recognized that Africa poses a difficult case for a pure diffusion theory particularly because of large family preferences [7].

Due to high fertility, the Government of Uganda promulgated its first explicit National Population Policy in 1995 but this was revised in 2008 due to persistent high fertility among other challenges [8]. In recognition of the health and economic benefits of family planning, Uganda increased its allocation for family planning supplies from US dollars 3.3 million in 2012 to 6.9 million in the financial year 2013/2014 [9]. In its Family Planning Costed implementation plan, 2015–2020; the government committed to reducing the unmet need for family planning to 10 percent and to increasing the modern contraceptive prevalence rate to 50 percent by 2050 [10]. Relatedly, Uganda Demographic and Health Survey (UDHS) aims to reduce fertility rates from 5.4 children per woman in the 2016 to 5.1 in 2019 [11].

While some studies had suggested that Uganda was one of the countries that experienced stalled fertility transitions, Kabagenyi et.al [12] demonstrated that the country is in the process of fertility transition but found no evidence of a stall. In 2017, with a total fertility rate (TFR) of 5.4, Uganda for the first time was not among the ten highest fertility countries in the world [13]. This demonstrates that although fertility in the country remains high, it is declining. Further demonstration of fertility decline is observed from the Uganda Population and Housing Census [14] and the Uganda Demographic and Health Surveys [15] conducted over time which have shown visible reductions in the country's fertility levels.

The fertility decline in Uganda has shown disparities among sub groups for instance faster decline is shown among the most educated women and those residing in urban areas and regions in the country [16, 17]. Although countries with higher educational levels have been associated with rapid fertility decline compared with their counterparts, this has not been the case in the East African region. For example, despite lower educational levels in Tanzania and Rwanda compared to Uganda, fertility in the two countries has shown more pronounced decline [18]. Education has been pointed out as one of the factors that have influenced fertility declines in urban Uganda [19]. Because completion of primary and transition to secondary has largely remained a prerogative of children from better socio-economic backgrounds and urban areas [20], the rural areas may lag behind urban areas in fertility reduction. Women in rural areas have higher fertility than women in urban areas (TFR of 5.9 versus 4.0 children). The TFR among women in rural areas declined from 7.1 in 2006 to 5.9 in 2016. In urban areas, the TFR has had a less consistent pattern, fluctuating around 4.0 [15]. Decomposition studies have quantified the contribution of women's social, economic, and demographic characteristics on fertility levels [21–23]. However, studies have not focused on sub groups. The analysis herein was done by decomposing fertility change in the period 2006 to 2016 among women aged 15–49 years in rural areas of Uganda.

## 2. Methods and Materials

**Figure 1:**
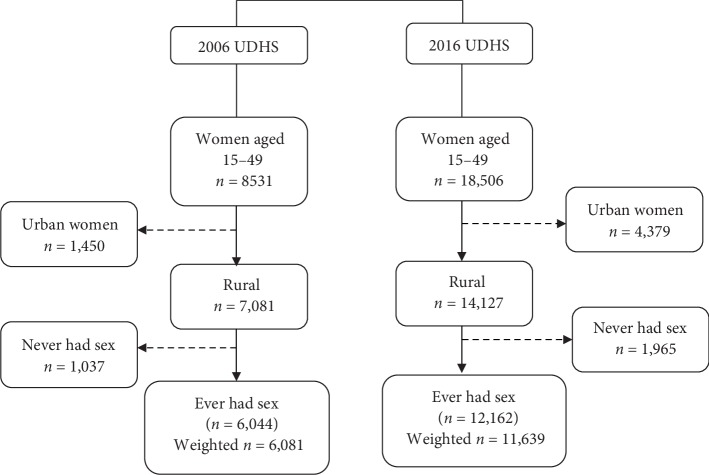
Derivation of the study sample.

Secondary data obtained from the year 2006 and 2016 Uganda demographic and health surveys were used in this study. The nationally representative cross-sectional surveys collected comparable demographic and health data on women aged 15–49 years. In both the years 2006 and 2016 UDHS, women aged 15–49 years were asked whether they had ever given birth and about the number of births they have ever had. Only never married, currently married/in union, and formerly married women who reported to have ever had sex were included in the current study. This inclusion criterion is based on the fact that naturally, women who have never had sex have no known exposure to the risk of pregnancy and childbirth. Although, most demographers consider marriage as the beginning of exposure to frequent sex and childbearing, studies in Africa have reported that pregnancy and childbearing may begin before marriage and a significant number of first births may occur before marriage [24–26]. We note that it is possible that there was under-reporting and misreporting on the question of sexual activity. Women who could have ever had sex but did not declare so were excluded from the study and this may lead to underestimation. This possibility of exclusion is most likely because questions on sexual activity are sensitive to young people and especially those who are unmarried in cultural and religious contexts where premarital sexual activity is frowned upon. A total of 6,044 and 12,612 women aged between 15 and 49 years who resided in rural areas in the years 2006 and 2016 were respectively selected from 8,531 and 18,506 women that were interviewed in the years 2006 and 2016 surveys. Although we mainly focus on the years 2006 and 2016 surveys, the 2011 data was also used to identify the trend in TFR during the 2006–2016 period. The data was first weighted using the technique for complex survey designs. A weighting variable was generated using the sample weight variable in the DHS data and was applied in all statistical commands. The weighted sample size for the two surveys were 6,081 and 11,639 respectively.  Figure 1 shows how the study sample was derived.

Our analysis takes the number of children ever born (CEB) to a female respondent in the two surveys as the dependent variable. CEB is a measure of the reported number of children born to a woman up to the moment at which the data was collected [4]. Other measures that can be used are TFR and general fertility rate (GFR). TFR is a synthetic measure that is based on hypothetical cohort of women of reproductive age with assumptions of constant birthrates over the lifespan and that no one will leave the hypothetical cohort [27]. On the other hand, GFR refers to the number of births per 1000 women of childbearing age [28]. It relates the number of births to the number of females in the reproductive age group and thus measures the general reproductive performance of the women per year [27]. It does not take into account the fact that within the range of the childbearing years for females of 15 to 49, there are differences in the extent to which the women produce children [4]. Both the GFR and TFR rely on current behavior (last 3 or 5 years), unlike the CEB. CEB was selected because it is a measure of actual cumulated fertility by the woman and the study intended to quantify the contribution of various factors to the variation in cumulated fertility of the women during the 2006–2016 period. In addition, because the study applied a nonlinear decomposition technique that deals with count outcomes such as the number of children, we found CEB to be a more suitable outcome. The independent variables were the age, education, residence, wealth quintile, type of family (having co-wives), working status of women, exposure to family planning messages, knowledge about contraceptives, current contraceptive use, age at first sex, ideal family size and age at first marriage. To cater to women that give birth before marriage, we introduced a category “not yet married”.

Statistical analysis was undertaken using STATA. Descriptive summaries indicating women's socio-demographic characteristics were performed using frequency distribution. Using the tfr2 tool [29], we generated and described the age specific fertility pattern and total fertility rate of the rural women during the 2006–2016 period. Secondly, because our outcome variable is a count of the CEB by a woman, a Poisson model was used to analyze factors associated with CEB. Poisson regression model is superior to ordinary least squares (OLS) or other linear models because the distribution of a count variable such as CEB which is a positive integer, is heavily skewed with a long right tail [30]. OLS is appropriate only if the dependent variable, and the count, is independently and identically distributed. Counts such as CEB are however nonlinear and thus application of the linear regression models which assume constant variance can result in inefficient, inconsistent and biased estimates.

At the bivariate level of analysis, Poisson regression of each independent variable and CEB offset by the natural logarithm of the current age for women was conducted for each survey year. The model was offset by the natural logarithm of the current age of the woman because age is highly correlated with CEB. This was intended to find out the factors associated with fertility of rural women in the respective surveys. The significant factors from this analysis were included in the Poisson decomposition model to quantify the contribution of the factors to the change in fertility among rural women during the 2006–2016 period. For ease of interpretation, we exponentiated the coefficients to yield the incident rate ratio (IRR). The IRR quantifies the direction and strength of the relation between the predictors and the CEB.An IRR value that is greater than 1 means higher likelihood of having children for a particular category of the independent variable compared to the reference category while that less than 1 implies lower fertility (reduced likelihood of giving births to children) for the category in comparison to the reference category.The IRR compares the rate of childbearing for a category of the rural women relative to their reference category and shows how changes in an explanatory variable affect the rate at which the outcome variable occurs. For example in Table 1 column 1 for education, the value of 0.901 for primary means that rural women who had attained primary level of education had lower fertility compared to their counterparts who had not attained any level of education. This implies that these women had 9.9% fewer children compared to their counterparts with no level of education. As CEB is highly correlated with current age of the women, in our model, current age was used as an offset term.

At the multivariate level, a nonlinear multivariate decomposition [31] technique that portions change over time into components attributable to changing characteristics of population and variation in effects of the characteristics on an outcome was applied. Before the decomposition analysis, multivariate Poisson regression of CEB was done separately for both survey year 2006 and 2016. The results are in Tables 2, 3 respectively. The decomposition model was used to partition the 2006–2016 change in CEB into components attributable to changing characteristics of women and variation in effects of the characteristics on CEB. Changing characteristics refers to part of the change in an outcome over time that is attributable to the changing composition of the group by selected characteristics and this is also called the characteristic effects. On the other hand, the variation in effects refers to the part of the differential attributable to differences in effect of the characteristics (coefficients) on the mean outcome and is also known as the coefficient effects [31]. In this study's context, the coefficient effects represent variations in the risk of childbearing that was observed during the 2006–2016 period. All the statistical significances of associations were determined at the 0.05 level of significance.

Since this study was an analysis of a secondary dataset that did not have personal identifiers, ethical approval was not necessary. We however sought permission to access and use the datasets from DHS through the link https://dhsprogram.com/data/available-datasets.cfm. The required access was subsequently permitted and the conditions for use of the data have been observed.

## 3. Results

The results presented in Table 4 are from a weighted sample size of 6,081 and 11,639 rural women in the 2006 and 2016 surveys respectively. The results indicate that in both the 2006 and 2016 samples, most of the rural women reported that they had attained a primary level of education and that relative to the 2006 sample, the 2016 sample had a higher proportion of rural women who had attained a secondary level and higher. The two survey samples had bigger proportions (45% in 2006 and 46% in 2016) of women from poor households. Relatedly, most of the rural women in the surveys reported that they were from male headed households and majority of them were currently working. In both the 2006 and 2016 surveys, the findings indicate that slightly more than half of the rural women did not have a co-wife but a quarter of the respondents in 2006 and 28.7% in 2016 reported that they were either single or were not sure whether their partners had other wives.

Rural women who reported having knowledge of family planning methods constituted the majority in both 2006 and 2016 samples. Furthermore the findings indicate that in both samples, at least half of the rural women reported being exposed to family planning messages through the mass media while majority were not currently using contraceptives. The findings indicate that most rural women reported that they had their first sexual intercourse when they were aged 15–19 years. More than half (54%) of the rural women in 2006 reported having had their first sexual intercourse aged 15–19. This proportion was 70% in 2016. It is also important to observe that in both 2006 and 2016 surveys, the proportion of the rural women whose age at sexual debut was younger than 15 years remained at 20.6% and the proportion that delayed having their first sex until age 20 years and older was 25% in 2006 and only 9% in 2016. This could be an indicator that the proportion of rural women that delay their first sexual intercourse after the adolescence period is reducing. Relatedly, the results indicate that the biggest proportion of rural women in both 2006 and 2016 reported their age at first marriage as 15–19 years but in 2016, the proportion that reported their age at first marriage as 20 years and older was higher than that of 2006. Regarding ideal family size, the results indicate that for both surveys, majority of the rural women preferred to have at least five children although the 2016 proportion was lower than that of 2006. The details are presented in Table 4.

### 3.1. Association between Selected Characteristics and Fertility of Rural Women in the 2006 and 2016 Surveys

The fertility of rural women was assessed on the basis of a Poisson model of CEB offset by the natural logarithm of the current age of the women aged 15–49 years in the two respective surveys. Table 1 presents results on fertility of rural women by selected characteristics in the two survey years. The IRR value is a quantification of the direction and strength of the association between predictors and CEB.IRR compares rates of childbearing for the rural women.An IRR value that is greater than 1 means that the rate of childbearing for a category of rural women was higher than that of the reference category while that less than 1 implies lower rate of childbearing compared to the reference category. The results show that in both 2006 and 2016, the rate of childbearing reduced with an increase in educational attainment. Rural women who had attained a secondary and higher level of education had significantly lower fertility (IRR of 0.589 and 0.508 in 2006 and 2016 respectively) than their counterparts who had not attained any level of education. Our findings also show that in both the 2006 and 2016 surveys, rural women from the households classified as rich had significantly lower fertility (IRR of 0.921 in 2006 and 0.823 in 2016) compared to their counterparts from poor households. The findings also show that women who reported being from female headed households had significantly lower fertility compared to their counterparts from male headed households. Regarding the working status of women, Table 1 shows that women who reported that they were currently working had a significantly higher IRR compared to their counterparts who were not. In both surveys, the fertility of the rural women who were currently working was about 15% higher than that of their nonworking counterparts. Furthermore, the results show that the fertility of rural women who were either single or not sure of whether their partner had other wives was significantly lower (IRR of 0.735 and 0.732 in 2006 and 2016 respectively) than that of their counterparts who did not have a co-wife. On the other hand, our findings show that in both surveys, women who had a co-wife had higher fertility relative to those who did not have. This could demonstrate the effect of polygamy on rural fertility but needs further investigation.

Table 1 results also show that in both 2006 and 2016, knowledge of any family planning methods did not show significant association with CEB. Relatedly, the findings show that in 2006, there was no relationship between exposure to family planning messages through mass media and fertility of the rural women but in 2016, the a significant association was observed as women who reported being exposed to family planning messages had significantly low fertility (IRR of 0.908) relative to that of their counterparts who were not exposed. Our findings show that paradoxically, in both surveys, women who were using contraceptives had a significantly higher fertility (IRR of 1.076 in 2006 and 1.136) compared to their counterparts who were not. This may be because in most cases it is high fertility women that use family planning methods. The findings also show that fertility of the rural women whose sexual debut was in the 15–19 years of age category, was relatively lower (IRR of 0.853 and 0.813 in 2006 and 2016 respectively) than that of whose sexual debut was in the age category of younger than 15 years. We observe that the IRR of rural women whose sexual debut was age 20 years and older equally were 0.919 and 0.577 in the 2006 and 2016 surveys respectively. This implies lower fertility among women who delay sexual debut relative to those whose sexual debut occurs below the age of 15 years. From the findings we also show that women who gave a nonnumeric response to the question of ideal number of children had the highest fertility compared to those whose ideal number of children was 0–2. The findings indicate that for both surveys, the fertility of rural women increased with the number of desired children. Finally, Table 1 results indicate that relative to women who were not yet married, the fertility of rural women significantly reduced with an increase in age at first marriage.

### 3.2. Change in Fertility among Women in Rural Areas of Uganda

**Figure 2:**
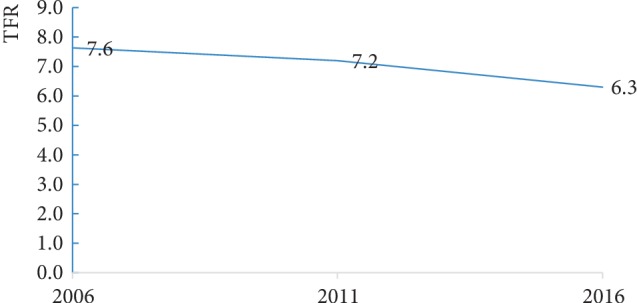
Total fertility rates of the rural women for the period 2006–2016.

Using the tfr2 tools, the results in Figure 2 indicate that the TFR of rural women reduced from 7.6 in 2006 to 6.3 children per woman in 2016. However, between the 2006–2016 period, the 2011 UDHS was conducted and the results show that rural women had a TFR of 7.2 children per woman.

**Figure 3:**
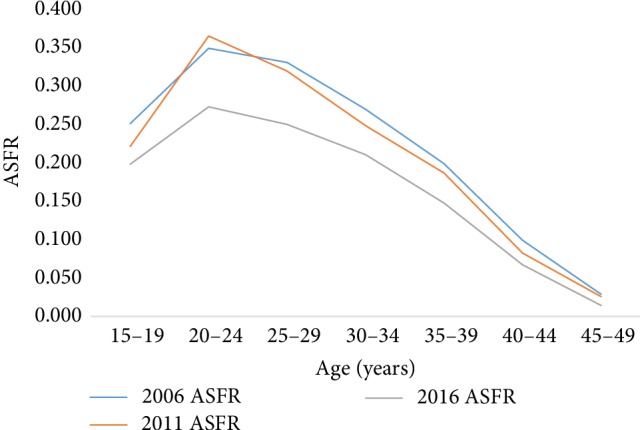
Age specific fertility rates of rural women for the period 2006–2016.

The results in Figure 3 indicate that the age specific fertility rates (ASFR) for the rural women in 2006 and 2011 were higher than those of their 2016 counterparts.

To determine whether the 2006–2016 change in fertility observed among rural women was significant, one-way analysis of variance (ANOVA) was conducted on CEB and the year of survey. The findings indicated that during the period 2006–2016, the fertility (MCEB) among women in rural areas significantly (*p* < 0.001) reduced from 4.5 in 2006 to 3.9 in 2016.

### 3.3. Decomposition of the Fertility Change among Rural Women

The results in Table 5(a) indicate the overall decomposition of change in fertility observed among rural women during the 2006–2016 period. The results indicate that 74% of the change in fertility among rural women can be attributed to variation in effects of the characteristics on CEB in the period 2006–2016 while 26% of the change was due to changing socioeconomic and demographic characteristics of women in the period. Variation in effects of the characteristics is the decomposition component that is attributable to differences in effect of the characteristics (coefficients) on the mean number of children ever born and is also known as the coefficients effects as labelled in the Tables 5(a), 5(b), and 6. The coefficient effects present changes in the risk of childbearing for the rural women of certain characteristics over time. Changing characteristics is the decomposition component that is attributable to changing composition of the group by selected characteristics and this has been labelled the “characteristics effects” in the tables. The percentage are derived by dividing the coefficient on each component by the sum of (Total) coefficients. For example, 26.4% on the characteristics effects is got from the ratio of −3.909 to the total of (−3.9091 + −10.880). This applies even for the detailed decomposition results in Table 6.

When current contraceptive use was dropped from the decomposition model, the contribution associated with changes in the composition of women increased to 42% while that associated with the effect of changing characteristics on mean level of fertility reduced to 58%. This signifies the importance of contraceptive use. Results in Table 5(b) are summary decomposition results when contraceptive use is excluded from the model.

While the results in Tables 5(a) and 5(b) are for the overall decomposition of the number of children ever born by the rural women in the 2006–2016 period. Table 6 shows the detailed decomposition results and indicates how much each category per selected characteristic contributed to the observed variation in fertility as measured by MCEB. The overall percent contribution of selected characteristic is arrived at by summing all the percentages on each category of the respective characteristic. As an example, for education, we add the values 1.4 and 19.2 that are in the fourth column of Table 6 to obtain 20.6% of the change in fertility that is attributable changing composition of the rural women by education attained between 2006 and 2016. The detailed decomposition results in Table 6 indicate that with respect to the categories of education attained, an increase in proportion of women who had attained at least secondary level of education can be associated with 19% of the change in fertility. The findings reveal that the slight increase in the proportion of rural women who belonged to the middle wealth category during the 2006–2016 period was associated with 0.2% of the reduction in fertility. The findings also indicate that change in proportion of women who were from female headed households can be associated with 0.4% of the change in fertility among the rural women during the period. The findings indicate that change in the proportion of rural women who were currently working at the time of the survey was associated with 3% of the reduction in fertility observed during the 2006–2016 survey period. Our findings also reveal that changes in the proportion of women who reported having a co-wife and their counterparts who were single/not sure of their co-wife status, were respectively associated with 1.3% and 1% of the fertility reduction among the rural women.

The study findings show that an increase in the proportion of rural women who reported being exposed to family planning messages through mass media was associated with 1.3% of the reduction on fertility that was observed among the rural women during the 2006–2016 period. The findings further indicate that between 2006 and 2016, the proportion of women who had sexual debut aged 15–19 years increased and this can be associated with 8.6% of the reduction in fertility of the rural women during the period. On the other hand, there was a reduction in the proportion of women whose age at sexual debut was 20+ years and this led to an increase in fertility (by 24.2%) that could have counteracted the gains in fertility reduction due to postponement of sexual debut. Thus the overall contribution of age at first sex to the change in fertility of the rural women was −15.5%. Similarly, Table 6 findings revealed that changes in composition of women by age at first marriage can be associated with an overall contribution to the change in fertility of 22.2%. More specifically, the observed reduction in percentage of rural women whose reported age at first marriage was below 15 years was associated with 26.2% of the reduction of fertility among the rural women while the reduction in the proportion of women whose age at first marriage was 15–19 years contributed 37.8% to the fertility decline observed during the period. In addition, the findings indicate that family size preference was associated with an overall contribution of 8.3% to the observed change in fertility among the rural women during the 2006–2016 period but specifically, the decrease in the proportion of women whose ideal number of children was 5+ children was associated with 8% of the observed change in fertility while the reduction in percentage of the rural women who gave a nonnumeric response to the question on ideal number of children was associated with 2.7% of the reduction in fertility.

Regarding the effects of the characteristics on the observed variation in fertility, only education level attained, wealth, age at first sex, sex of household head and polygamy were significant contributors to the observed change in fertility. The findings indicate that differences in the risk of childbearing was associated with an overall contribution of 51.5% of the observed change in fertility among rural women. The differential effects in the 2006–2016 risks of childbearing for rural women who had attained primary and secondary levels of education was associated with 36.5% and 13.5% respectively to the observed change in fertility. The findings further indicate that 13.4% of the observed change in fertility can be associated with the 2006–2016 variation in effect of wealth (specifically being rich) on fertility. Relatedly, the variation in risk of childbearing among rural women from female headed households during the 2006–2016 period contributed 12.3% to the change in fertility that was observed. Our findings in Table 6 also indicate that increased fertility among women in polygamous unions was associated with a 10.7% increase in overall fertility. Finally, the results indicate that difference in the risk of childbearing among rural women whose age at first sex was 20+ years was associated with 47.8% of the change in fertility among the rural women.

The results in column 1 of Table 6 are based on the MCEB of the various categories of the variables compared to the reference category. For instance, with respect to education. The −0.207 indicates that between 2006 and 2016, the MCEB for the rural women who had attained primary level of education was 20.7% below the MCEB for the “no education” group of rural women.

## 4. Discussion

Our findings indicated the importance of female education in fertility transition. Both components of the decomposition showed that change in education had a small contribution to the observed variation in fertility between the 2006 and 2016 surveys. It is important to note however that the change in risk of childbearing (variations in the coefficients) for rural women with primary and secondary levels of education was associated with bigger percentage contribution to the observed change in fertility compared to the changes in the composition of women by education attainment. This implies that education in a way influences the fertility behavior of women in terms of deciding when to give birth and how many children to give birth to. Education is widely known to strongly influence women's fertility by delaying age at first marriage and reducing family size. The findings of this study partly agree with earlier studies [19, 22] found that increased education attainment by women was responsible for at least half of the fertility decline in sub Saharan Africa. Similarly, Jain & Ross, (2012) observed the transition from higher to lower fertility is associated with improved female education [32]. In Uganda, it was found that female education, especially attainment of at least secondary education level increases the likelihood of using contraceptives and reduces fertility [33]. Education was also found to have significant influence on fertility in other countries. Sharma (2015) asserted that fertility of Nepal can be reduced significantly by slightly increasing the educational status of women [34]. Shapiro & Gebreselassie (2008), Westoff, Bietsch, & Koffman (2013), and Shakya & Gubhaju (2016) also observed that increasing women's educational attainment is a key factor contributing to sustained fertility decline [35–37].

Although, the change in composition by sex of household head did not show a significant contribution to the observed change in fertility, household headship significantly contributed to the behavioral component of the decomposition model. This may be linked to fertility decision making in households. Women from female headed households are more likely to take independent decisions regarding fertility. This may not be the case with women from male headed households especially in rural areas where there is limited empowerment. This points to the fact that fertility decisions may be taken by the household head who in most cases is a male.

Our findings indicate that in both 2006 and 2016, women who reported that they were currently working had a higher MCEB than their counterparts. Furthermore, although the proportion of rural women who were currently working in the 2006 survey was slightly higher than that of 2016, the findings indicate that this variation made a significant contribution to the observed change in fertility. Our findings indicate that women's working status contributed to 1.9% of the observed variation in fertility. This finding is partly in line with what other studies have reported about the importance of female employment in fertility transition. In Botswana, a study found that nonworking mothers had more number of children ever born than their working counterparts [38]. Relatedly, in women's participation in labor force was found to reduce fertility rates [39]. In Poland, work was found to have a direct effect on the number of births with women who work having more children than their counterparts who did not work [3].

In sub-Saharan Africa, the age at first marriage has been found to be more instrumental in influencing fertility changes [40]. Kabagenyi et al. (2015) attributed Uganda's persistent high fertility to a young age at marriage that has remained considerably low [12]. In countries like Colombia, Dominican Republic, and Turkey, a decline in the median age at marriage was followed by increase in fertility [41]. The postponement of marriage contributed to the reduction of fertility in some countries over the 1990–2008 period [42]. In an earlier study conducted on change in fertility among women in Uganda [23], the age at first marriage was one of the biggest contributors to the variation in fertility observed between 2006 and 2011. Our current study indicates that the proportion of surveyed rural women who reported their age at first marriage as 20 years and older increased between the two survey years and this contributed a significant percentage to the overall change in fertility observed in rural areas. Considering that the utilization of contraceptives in Uganda's rural areas is still lower than in the urban areas, the age at first marriage in these areas continues to be a significant factor in the rural areas. This calls for efforts to improve accessibility to family planning services that not only provide contraceptive choices but rather target sensitization and education of the rural people about dangers of early marriage and the socioeconomic, health and demographic benefits that accrue from delayed marriage.

The findings in Table 6 indicated that the 2006–2016 change in fertility was significantly associated with the variation in the women's preferred number of children in the two surveys. Family size preferences affect people's fertility behaviors and especially decisions on whether to use or not to use fertility control measures such as contraceptives. These findings support the view [17, 41, 43–45] which have noted the importance of shift in desired family size in fertility decline in a number of countries.

Exposure to mass media is among the factors that determine the number of children desired and increased the use of modern contraceptives especially as they relay family planning messages [36, 45, 46]. The diffusion of information about methods of birth control is now considered an important mechanism of fertility change [1]. Our findings show that exposure to family planning messages through the mass media made a small contribution of 0.9%. Although, the percentage contribution seems to be small, it is important to note that mass media coverage for rural areas is equally low as most people have limited or no access to radios and televisions. There is no doubt that increased uptake of family planning would lead to significant declines in unwanted fertility. It is imperative that more investments in family planning programs are made to strengthen existing programs so that they can reach even the hard-to reach areas in the rural Uganda.

Sexual debut undoubtedly plays a significant role in fertility transitions. Delayed sexual debut implies delayed exposure to pregnancy and childbearing. In many African societies, first births precede formal marriage and in some cases proof of fecundity is an important precondition to formalizing the marriage bond [25]. Our findings show that age at sexual debut was a significant contributor to the observed reduction in fertility. The study finds that although change in the proportions of women by their age at first sexual intercourse made relatively small percentage contributions to the overall change in fertility among the rural women, age at first sex had the second biggest percentage contribution on the coefficient effects of the decomposition. The findings revealed that the reduction in the risk of childbearing associated with age at first sex for the rural women during the 2006–2016 period significantly contributed to the observed reduction in fertility. The type of family (whether women had a co-wife or not or were still single) contributed to the reduction in fertility. With the proportion of women who reported having co-wives decreasing from 22.1% to 19.1 in the 2006–2016 period and those who were single increasing from 25.4 to 28.7% in the period, the results showed that this contributed 1.6% to the reduction in fertility. Polygamy which is largely a common practice in the rural areas but appears to be changing could have contributed to this observation. The effect of polygamy on the fertility behavior of women has contributed 23% to the observed reduction in fertility as indicated in Table 6. A study should be done to explore the influence of polygamy on the fertility behavior especially in rural areas.

The strength of this manuscript is that the analysis is based on survey data which is nationally representative. The DHS adheres to standard international protocols and processes to conduct surveys. Furthermore, the analysis technique used facilitates the portioning of change in an outcome over time into components attributable to changing socioeconomic and demographic composition of women and changing fertility behaviors.

Our analysis only included women who had ever had sex as they were the only ones with known exposure to pregnancy and child birth. This inclusion was based on women who answered the question on their sexual activity. This question of sexual activity is sensitive in most rural areas as unmarried people are expected to abstain from sex until they are married. With this in mind, we note that underreporting and/or misreporting are possible and thus some women who had ever had sex but did not declare so could have been excluded. Whereas, the 2011 UDHS was conducted between 2006 and 2016, our analysis technique is limited to analyzing differences between two groups only. Thus, the decomposition technique did not allow inclusion of more than two surveys. We opted for two surveys that have a relatively longer interval to enable us to analyze changes in fertility since such changes are known to take longer periods. Thus, we pooled the 2006 and 2016 datasets to conduct a decomposition analysis of change in fertility of rural women over a 10-year period.

Although DHS are generally a good source of data for fertility analysis, it is noteworthy that the fertility data are prone to recall issues [47] and the nature of birth history questions makes it possible for women to backdate births in order to avoid completing the birth history questions [26]. Furthermore, we note that DHS data on fertility may not be of sufficient quality to examine trends in fertility especially when using two data points [48] but our study is aimed at quantifying the factors contributing to the difference in fertility levels of rural women using the years 2006 and 2016 rather than to examine trends. In addition, our analysis relied on cross-sectional surveys and hence we only find associations and not causal relationships. The study quantified the contribution of the factors associated with change in fertility of rural women observed in the 2006–2016 period.

## 5. Conclusion and Implications

The change in fertility observed among women who resided in rural areas during the period 2006–2016 is attributable to both changing composition of women by socioeconomic and demographic characteristics and changing fertility behavior. Education level attained and age at first sex showed significant contributions on both components of the decomposition. Other factors that contributed to the observed reduction in fertility were; sex of household head, working status of women, exposure to family planning messages, contraceptive use, family size preference and age at first sex.

Continued improvements in access, attendance and completion of secondary schools by women in rural areas will be key drivers to Uganda's overall transition to low fertility. Furthermore, with improved access to mass media in the rural areas, there can be changes in attitudes and large family size preferences which can create a conducive environment for the utilization of family planning services in the rural communities. Efforts should therefore focus on applying appropriate methods to deliver appropriately packaged family planning messages to these communities.

Our findings have pointed to the fact that age at first sex is an important determinant of fertility in Uganda's rural areas. The government of Uganda has for long sponsored campaigns and messages on sexual abstinence among young people in Uganda but this has largely been for HIV prevention. Such campaigns need to be made more comprehensive so that issues of early and frequent childbearing can be incorporated.

## Figures and Tables

**Table 1:** Factors associated with fertility of rural women in 2006 and 2016.

Characteristic	2006				2016		
	IRR	*P*-value	95% CI	IRR	*P*-value	95% CI
*Education level*						
No education	1.000			1.000		
Primary	0.901	*0.001*	0.874–0.928	0.810	*0.001*	0.789–0.832
Secondary+	0.589	*0.001*	0.553–0.628	0.508	*0.001*	0.488–0.528

*Wealth*						
Poor	1.000			1.000		
Middle	1.003	0.858	0.967–1.041	0.977	0.111	0.950–1.005
Rich	0.921	*0.001*	0.889–0.953	0.823	*0.001*	0.800–0.848

*Sex of household head*						
Male	1.000			1.000		
Female	0.933	*0.001*	0.903–0.964	0.893	*0.001*	0.870–0.916

*Current working status*						
Not working	1.000			1.000		
Working	1.151	*0.001*	1.089–1.216	1.157	*0.001*	1.115–1.201

*Polygamy*						
No cowife	1.000			1.000		
Has cowife	1.040	*0.013*	1.009–1.073	1.120	*0.001*	1.092–1.148
Single/not sure	0.735	*0.001*	0.702–0.768	0.732	*0.001*	0.709–0.757

*Knowledge of any family planning methods*						
No knowledge	1.000			1.000		
Has knowledge	1.042	0.175	0.982–1.105	0.884	0.405	0.662–1.181

*Exposure to family planning messages*						
Not exposed	1.000			1.000		
Exposed	0.974	0.078	0.946–1.003	0.908	*0.001*	0.886–0.930

*Contraceptive use*						
Not using	1.000			1.000		
Using	1.076	*0.001*	1.041–1.112	1.136	*0.001*	1.111–1.163

*Age at first sex*						
Below 15	1.000			1.000		
15–19	0.853	*0.001*	0.822–0.885	0.813	*0.001*	0.793–0.834
20+	0.919	*0.001*	0.882–0.956	0.577	*0.001*	0.549–0.607

*Family size preferences*						
0–2	1.000					
3–4'	1.282	*0.001*	1.155–1.423	1.178	*0.001*	1.093–1.270
5+	1.753	*0.001*	1.584–1.940	1.737	*0.001*	1.614–1.869
Nonnumeric	1.654	*0.001*	1.465–1.868	1.941	*0.001*	1.782–2.113

*Age at first marriage*						
Not yet married	1.000			1.000		
Below 15	5.622	*0.001*	4.762–6.638	5.534	*0.001*	4.929–6.214
15–19	4.796	*0.001*	4.069–5.654	4.468	*0.001*	3.986–5.009
20+	3.993	*0.001*	3.378–4.720	3.734	*0.001*	3.325–4.194

**Table 2:** Multivariate Poisson regression results for the 2006 survey.

Variable	IRR	*P*-value	95% CI
*Education level*			
No education	1.000		
Primary	0.933	*0.001*	0.905–0.961
Secondary+	0.764	*0.001*	0.721–0.809

*Wealth*			
Poor	1.000		
Middle	1.025	0.167	0.990–1.061
Rich	1.025	0.149	0.991–1.059

*Sex of household head*			
Male	1.000		
Female	1.025	0.152	0.991–1.060

*Current working status*			
Not working	1.000		
Working	1.057	*0.016*	1.010–1.107

*Polygamy*			
No cowife	1.000		
Has cowife	1.026	0.103	0.995–1.059
Single/not sure	0.907	*0.001*	0.870–0.946

*Knowledge of any family planning methods*			
No knowledge	1.000		
Has knowledge	1.101	*0.002*	1.035–1.172

*Exposure to family planning messages*			
Not exposed	1.000		
Exposed	0.993	0.602	0.966–1.020

*Contraceptive use*			
Not using	1.000		
Using	1.132	*0.001*	1.100–1.166

*Age at first sex*			
Below 15	1.000		
15–19	0.943	*0.002*	0.910–0.978
20+	0.942	*0.002*	0.907–0.979

*Family size preferences*			
0–2	1.000		
3-4'	1.132	*0.005*	1.038–1.238
5+	1.411	*0.001*	1.296–1.537
Nonnumeric	1.374	*0.001*	1.234–1.530

*Age at first marriage*			
Not yet married	1.000		
Below 15	4.121	*0.001*	3.479–4.881
15–19	3.708	*0.001*	3.144–4.372
20+	3.187	*0.001*	2.700–3.763

**Table 3:** Multivariate poisson regression results for the 2016 survey.

*Variable*	IRR	*P*-value	95% CI
*Education level*			
No education	1.000		
Primary	0.870	*0.001*	0.849–0.892
Secondary+	0.685	*0.001*	0.660–0.711

*Wealth*			
Poor	1.000		
Middle	1.021	0.081	0.997–1.046
Rich	0.982	0.160	0.957–1.007

*Sex of household head*			
Male	1.000		
Female	0.983	0.170	0.959–1.007

*Current working status*			
Not working	1.000		
Working	1.062	*0.001*	1.032–1.093

*Polygamy*			
No cowife	1.000		
Has cowife	1.084	*0.001*	1.060–1.109
Single/not sure	0.956	*0.003*	0.927–0.985

*Knowledge of any family planning methods*			
No knowledge	1.000		
Has knowledge	0.998	0.990	0.777–1.283

*Exposure to family planning messages*			
Not exposed	1.000		
Exposed	0.971	*0.005*	0.951–0.991

*Contraceptive use*			
Not using	1.000		
Using	1.146	*0.001*	1.124–1.168

*Age at first sex*			
Below 15	1.000		
15–19	0.906	*0.001*	0.883–0.929
20+	0.762	*0.001*	0.26–0.799

*Family size preferences*			
0–2	1.000		
3–4	1.077	*0.016*	1.014–1.145
5+	1.382	*0.001*	1.301–1.468
Nonnumeric	1.514	*0.001*	1.407–1.628

*Age at first marriage*			
Not yet married	1.000		
Below 15	3.726	*0.001*	3.319–4.182
15–19	3.369	*0.001*	3.013–3.768
20+	3.102	*0.001*	2.771–3.472

**Table 4:** Distribution of rural women by selected characteristics in 2006 and 2016.

Variable	2006		2016	
	Frequency (*n*)	Percent (%)	Frequency (*n*)	Percent (%)
*Age*				
15–19	679	11.2	1506	12.9
20–24	1253	20.6	2520	21.7
25–29	1139	18.7	2058	17.7
30–34	1035	17.0	1829	15.7
35–39	811	13.3	1506	12.9
40–44	643	10.6	1251	10.8
45–49	522	8.6	968	8.3

*Education level*				
No education	1507	24.8	1497	12.9
Primary	3766	61.9	7446	64.0
Secondary+	808	13.3	2696	23.2

*Wealth quintile*				
Poor	2,750	45.2	5,347	45.9
Middle	1,321	21.7	2,650	22.8
Rich	2,011	33.1	3,642	31.3

*Sex of household head*				
Male	4389	72.2	8089	69.5
Female	1693	27.8	3550	30.5

*Current working status*				
Not working	671	11.0	2252	19.4
Working	5410	89.0	9386	80.7

*Polygamy*				
No cowife	3192	52.5	6075	52.2
Has cowife	1344	22.1	2218	19.1
Single/Not sure	1545	25.4	3345	28.7

*Knowledge of any family planning methods*				
No knowledge	164	2.7	38	0.3
Has knowledge	5918	97.3	11601	99.7

*Exposure to family planning messages*				
Not exposed	2523	41.5	3816	32.8
Exposed	3559	58.5	7823	67.2

*Contraceptive use*				
Not using	4880	80.2	7729	66.4
Using	1201	19.8	3909	33.6
*Age at first sex*				
Below 15	1255	20.6	2398	20.6
15–19	3299	54.2	8164	70.2
20+	1528	25.1	1070	9.2

*Family size preference*				
0–2	339	5.6	642	5.5
3-4'	2338	38.5	5171	44.4
5+	3161	52.0	5509	47.3
Nonnumeric	243	4.0	317	2.7

*Age at first marriage*				
Not yet married	520	8.6	1303	11.2
Below 15	962	15.8	1415	12.2
15–19	3585	58.9	6192	53.2
20+	1015	16.7	2728	23.4

**Table 5:** 

(a) Overall decomposition (contraceptive use included)				
Component	Coefficient	STE	*P*-value	%
Characteristics effects	−3.909	0.796	0.001	26.4
Coefficient effects	−10.880	1.410	0.001	73.6

*Total*	−14.789	1.145	0.001	100.0

(b) Overall decomposition (contraceptive use dropped)				

Characteristics effects	−6.288	0.649	0.001	42.5
Coefficient effects	−8.501	1.324	0.001	57.5

*Total*	*−14.789*	*1.154*	*0.001*	*100.0*

**Table 6:** Detailed decomposition of the observed change in fertility among rural women based on MCEB.

Characteristics effects					Coefficient effects			
Variable	Coef × 1000	STE	*P*-value	%	Coef × 1000	STE	*P*-value	%
*Education level*								
No education	1.000				1.000			
Primary	−0.207	0.026	*0.001*	1.4	−5.392	1.644	*0.001*	36.5
Secondary+	−2.834	0.228	*0.001*	19.2	−2.054	0.636	*0.001*	13.9

*Wealth*								
Poor	1.000							
Middle	0.027	0.010	*0.009*	−0.2	−0.099	0.601	0.869	0.7
Rich	0.001	0.018	0.956	0.0	−1.974	0.944	*0.036*	13.4

*Sex of household head*								
Male	1.000							
Female	−0.056	0.027	*0.036*	0.4	−1.814	0.781	*0.020*	12.3

*Current working status*								
Not working	1.000				1.000			
Working	−0.450	0.102	*0.001*	3.0	0.497	3.175	0.876	−3.4

*Polygamy*								
No cowife	1.000				1.000			
Has cowife	−0.188	0.030	*0.001*	1.3	1.577	0.580	*0.007*	−10.7
Single/not sure	−0.153	0.043	*0.001*	1.0	1.571	0.880	0.074	−10.6

*Exposure to family planning messages*								
Not exposed	1.000				1.000			
Exposed	−0.187	0.074	*0.011*	1.3	−2.356	1.339	0.078	15.9

*Age at first sex*								
Below 15	1.000				1.000			
15–19	−1.268	0.178	*0.001*	8.6	−2.893	1.648	0.079	19.6
20+	3.583	0.215	*0.001*	−24.2	−7.071	1.315	*0.001*	47.8

*Family size preferences*								
0–2	1.000				1.000			
3–4	0.339	0.152	*0.026*	−2.3	−2.323	2.715	0.392	15.7
5+	−1.177	0.133	*0.001*	8.0	−1.209	3.598	0.737	8.2
Nonnumeric	−0.406	0.044	*0.001*	2.7	0.524	0.342	0.126	−3.5

*Age at first marriage*								
Not yet married	1.000				1.000			
Below 15	−3.869	0.219	*0.001*	26.2	−2.065	2.342	0.378	14.0
15–19	−5.585	0.320	*0.001*	37.8	−7.269	8.461	0.390	49.2
20+	6.112	0.339	*0.001*	−41.3	−0.591	2.270	0.795	4.0

## Data Availability

The datasets used for this study are publicly available through the link https://dhsprogram.com/data/available-datasets.cfm.
